# Hybrid Amyloid–Boron Nitride Aerogels for Lightweight Sound Insulation

**DOI:** 10.1002/adma.74088

**Published:** 2026-07-17

**Authors:** Mohammad Peydayesh, Andrin Lustgarten, Stefan Schoenwald, Enrico Boschi, My Hanh Bui, Ali Miserez, Felix Donat, Christoph R. Müller, Raffaele Mezzenga

**Affiliations:** ^1^ ETH Zurich Department of Health Sciences and Technology Zurich Switzerland; ^2^ Swiss Federal Laboratories For Materials Science and Technology (Empa) Duebendorf Zurich Switzerland; ^3^ University of Copenhagen Plant Glycobiology Frederiksberg C Denmark; ^4^ Centre For Sustainable Materials (SusMat) School of Materials Science and Engineering Nanyang Technological University Singapore Singapore; ^5^ School of Biological Sciences Nanyang Technological University Singapore Singapore; ^6^ Department of Mechanical and Process Engineering ETH Zürich Zürich Switzerland; ^7^ ETH Zurich Department of Materials Zurich Switzerland

**Keywords:** aerogel, amyloid fibrils, boron nitride, hybridization, self‐assembly, sound shielding

## Abstract

Boron nitride (BN) is a lightweight, thermally stable, and chemically inert material widely used in electronics and insulation, yet its synthesis typically relies on energy‐intensive, high‐temperature routes. Here, we introduce a sustainable, bottom‐up strategy to fabricate hybrid boron nitride–amyloid fibril (BNAF) aerogels, in which whey protein amyloid fibrils serve as renewable scaffolds that assist the organization of BN‐rich domains. A mild ultrasonication–freeze‐drying process yields ultralight aerogels with hierarchical porosity, increased BN‐like ordering relative to the protein‐free control, and fibril‐induced reinforcement. The hybrid aerogels combine mechanical reinforcement, thermal stability under moderate‐use conditions, and strong acoustic performance, achieving sound absorption coefficients above 0.8, noise reduction coefficients up to 0.63, and sound transmission losses of 25–30 dB. This combination of low density and strong sound shielding compares favorably with common lightweight acoustic materials such as fiberglass, establishing amyloid‐assisted BN–protein hybrid aerogels as promising candidates for moderate‐temperature, weight‐sensitive noise‐reduction applications.

## Graphical abstract

Amyloid fibrils template the mild formation of boron nitride–rich hybrid aerogels with hierarchical porosity, enhanced BN‐like ordering, and fibril‐reinforced mechanics. These ultralight materials combine thermal stability with strong sound absorption and transmission loss, offering a sustainable bio‐assisted route toward lightweight acoustic shields for moderate‐temperature, weight‐sensitive applications.

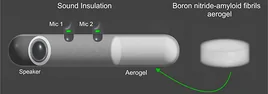

## 1 Introduction

Boron nitride (BN) is a multifunctional material with outstanding thermal stability, electrical insulation properties, and chemical inertness [1]. Structurally similar to graphite, hexagonal BN (h‐BN) consists of atomically flat layers of alternating boron and nitrogen atoms held together by van der Waals forces [2]. This structure gives rise to various nanostructures, ranging from 0D quantum dots [3] and 1D nanowhiskers [4] to 2D nanosheets [5] and 3D layered materials [6].

Owing to its high thermal conductivity (∼751 W/m·K for monolayer h‐BN at room temperature [7]), low thermal expansion, and dielectric properties, h‐BN is widely used in electronic applications as a thermal interface material or electrical insulator [8]. It plays a critical role in heat management for high‐power and high‐frequency devices [9], including semiconductors [10], LEDs, and power electronics [11]. Its structural stability at temperatures up to 1000 °C in air and 1200 °C in vacuum further enhances its suitability for demanding environments [12].

Beyond electronics, h‐BN is employed in protective coatings due to its hardness and wear resistance [13], and in water purification, where functionalized BN adsorbents effectively capture heavy metals and organic pollutants [14]. In cosmetics, it is used as a skin‐friendly additive, valued for its silky texture and oil‐absorbing properties [15], highlighting the material's broad applicability across industries.

h‐BN can be synthesized using either bottom‐up or top‐down approaches, each with distinct advantages and limitations. Among the bottom‐up methods, chemical vapor deposition (CVD) is widely used for producing high‐quality h‐BN films with controlled thickness and properties, by reacting boron and nitrogen precursors at temperatures of up to ∼1200°C [16]. Hydrothermal methods offer a lower‐temperature alternative (150–200°C) [17, 18], enabling scalable production of nanostructured h‐BN, such as nanosheets and nanoparticles, with tunable size and crystallinity.

Top‐down synthesis typically involves exfoliating bulk h‐BN crystals into thin layers via ultrasonication in solvents [19, 20]. Ball‐milling under pressure has also been explored as an energy‐efficient, room‐temperature route [21]. In all cases, parameters such as precursor choice, temperature, and pressure critically influence crystallinity, morphology, and stability [10]. Current synthesis techniques still face challenges, including high energy requirements, oxidation at elevated temperatures, non‐uniformity, and scalability limitations [13, 22, 23].

Boric acid (H_3_BO_3_) is a common boron source for BN synthesis, decomposing to boron oxide (B_2_O_3_) at high temperatures (∼330°C) [24] or via mechanochemical processes. Boron oxide readily reacts with nitrogen‐containing precursors to form BN [15]. Melamine (C_3_N_6_H_6_), containing 66 wt% nitrogen, and urea (CH_4_N_2_O) are commonly used as nitrogen precursors, due to their efficiency in BN formation and reduced need for excess reagents [8]. In particular, the combination of boric acid with melamine has become a common strategy, facilitating efficient BN synthesis and the formation of diverse nanostructures [25].

BN has been explored for hybridization with proteins for biomedical applications, including drug delivery [26], biosensing [27], and tissue engineering [28]. Such hybridization typically occurs through non‐covalent interactions or covalent functionalization. For example, Wang et al. produced h‐BN nanosheets by ultrasonication of bulk h‐BN in the presence of soy protein isolate, where hydrogen bonding, π–π interactions, and hydrophobic forces promoted protein–BN affinity [23]. However, in these applications, proteins mainly serve a bio‐compatibilization or dispersion role, whereas supramolecular protein assemblies such as amyloid fibrils can additionally provide high‐aspect‐ratio structural scaffolds for organizing hybrid inorganic–organic networks. Amyloid fibrils are elongated protein aggregates representing the lowest‐energy state in the protein folding landscape [29]. Artificial amyloids from inexpensive food protein precursors can form when the starting proteins are denatured and hydrolyzed at high temperatures (∼90°C) and acidic conditions (pH 2), producing peptides that self‐assemble into semi‐flexible fibrillar colloids [30]. Rich in β‐sheets and strands, amyloid fibrils typically measure only a few nanometers in width and tens of micrometers in length, giving them a very high aspect ratio and high surface‐to‐volume ratio [31] and extraordinary mechanical properties [32, 33]. As a result, amyloid fibrils have been applied in biomedical [34], environmental, and energy applications [35], only to cite a few examples.

Recent advances in ceramic and graphene‐based nanofibrous sponges and aerogels have shown that architected porous frameworks can combine ultralow density with exceptional mechanical robustness, thermal insulation, and acoustic damping [36]. For instance, Wang et al. reported blow‐spun ceramic nanofiber sponges that are ultralight, scalable, and resilient at high temperature, while maintaining recoverable compressibility and low thermal conductivity [37]. Jia et al. developed highly compressible, anisotropic lamellar ceramic sponges with superior thermal insulation and sound absorption performance [38]. Hierarchical ultralight graphene oxide aerogels prepared by emulsion freeze‐casting have also achieved broadband sound absorption by tailoring multiscale pore architectures [39]. These developments underscore the potential of aerogel‐type porous media for lightweight sound insulation, while motivating the exploration of more sustainable, bio‐templated scaffolds that can deliver comparable acoustic performance under moderate‐temperature conditions.

Hybridizing amyloid fibrils with BN offers the potential to develop novel, complex functional aerogels. In this work, BN–amyloid fibril hybrids (BNAF) are produced through a bottom‐up, low‐energy ultrasonication approach using boric acid and melamine as precursors. Amyloid fibrils act as scaffold‐mediating agents that assist the formation, dispersion, and organization of BN‐rich domains under mild processing conditions, providing a potentially sustainable route toward lightweight BN–protein hybrid aerogels. To showcase a possible application, the resulting materials are explored for sound‐insulating shields.

## 2 Results and Discussions

### 2.1 Hybrid BN‐Amyloid Fibrils Aerogels Fabrication and Characterization

**FIGURE 1:**
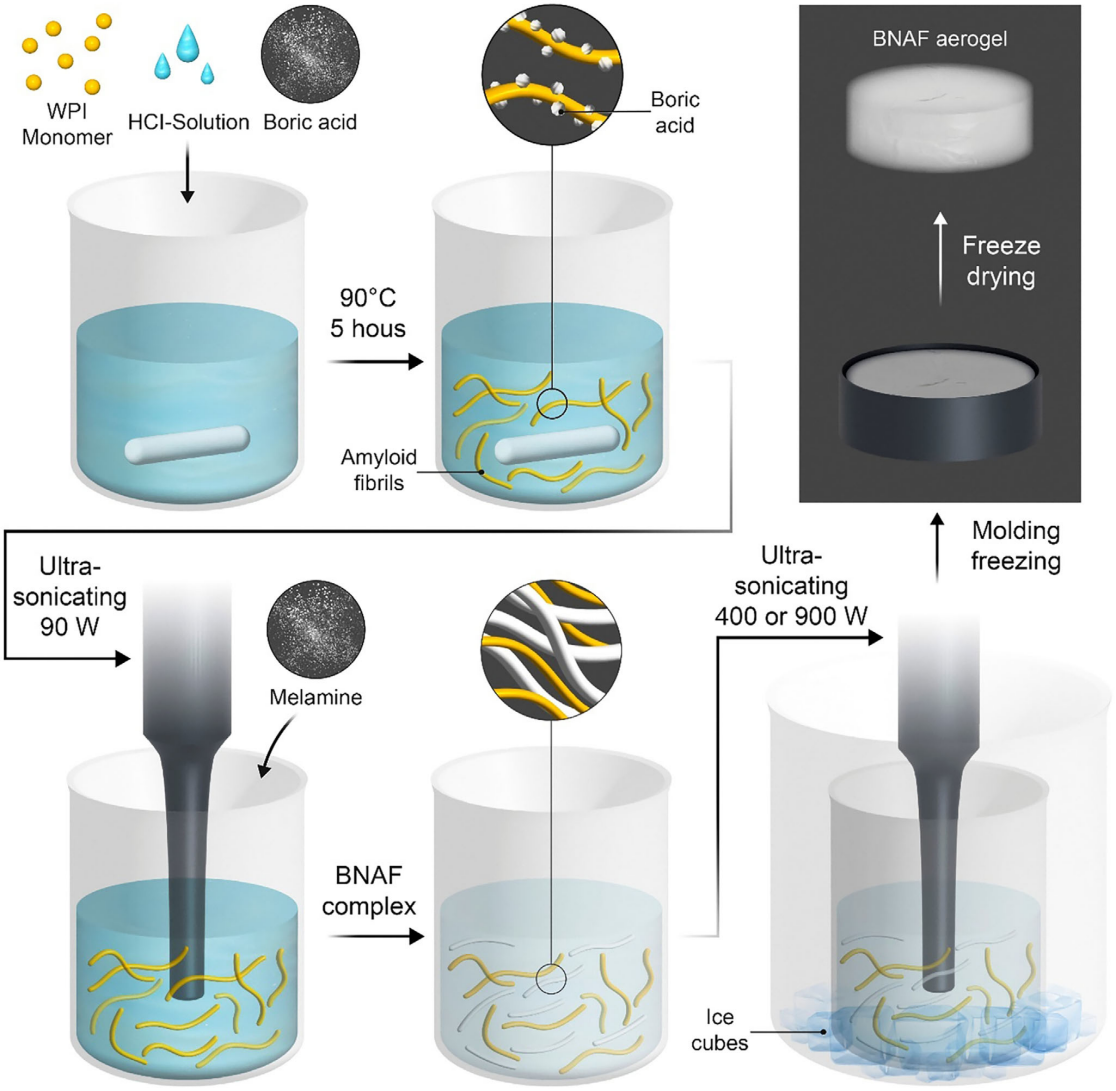
Schematic representation of the preparation process for BNAF materials, illustrating amyloid fibril formation (yellow fibrils), formation of BN‐rich hybrid domains (white fibrils) via melamine addition and ultrasonication, and subsequent aerogel fabrication through freeze‐drying.

The hybrid BN–amyloid fibril aerogels were fabricated through a multi‐step process involving amyloid fibril formation, incorporation of boric acid, melamine‐assisted BN synthesis, and subsequent aerogel structuring via ultrasonication and freeze‐drying (Figure 1). In the first stage, whey protein isolate (WPI) monomers were thermally converted into amyloid fibrils in the presence of boric acid, favoring the formation of stable boric acid–fibril complexes. This step likely promotes association of boric acid/borate species with the fibril surface through hydrogen bonding and interactions with hydroxyl, carboxyl, amide, and other polar groups present in the protein‐derived fibrils. The absence of visible boric acid re‐sedimentation after cooling supports stabilization of boric acid‐containing species in the fibrillar solution. Upon addition of melamine, a nitrogen‐rich precursor is introduced into this boron‐containing fibrillar environment. Ultrasonication then provides intense local mixing, cavitation, and transient high‐energy microenvironments, which may promote dehydration/condensation and organization of boron‐ and nitrogen‐containing species into BN‐like domains in close association with the fibrillar network, yielding a homogeneous BN–amyloid fibril hybrid suspension. We therefore propose that the amyloid fibrils influence this process by concentrating boron‐containing species near the fibril surface, providing a high‐aspect‐ratio scaffold for organizing inorganic domains, and stabilizing the resulting BN‐rich hybrid structures against uncontrolled aggregation. We emphasize, however, that the exact atomistic nucleation mechanism is not resolved in the present study and would require dedicated time‐resolved or surface‐sensitive characterization, so that future work should be devoted to elucidate in full the mechanisms. Finally, a high‐power ultrasonication and freeze‐drying sequence transformed this suspension into lightweight, porous aerogels suitable for further characterization and application testing.

**FIGURE 2:**
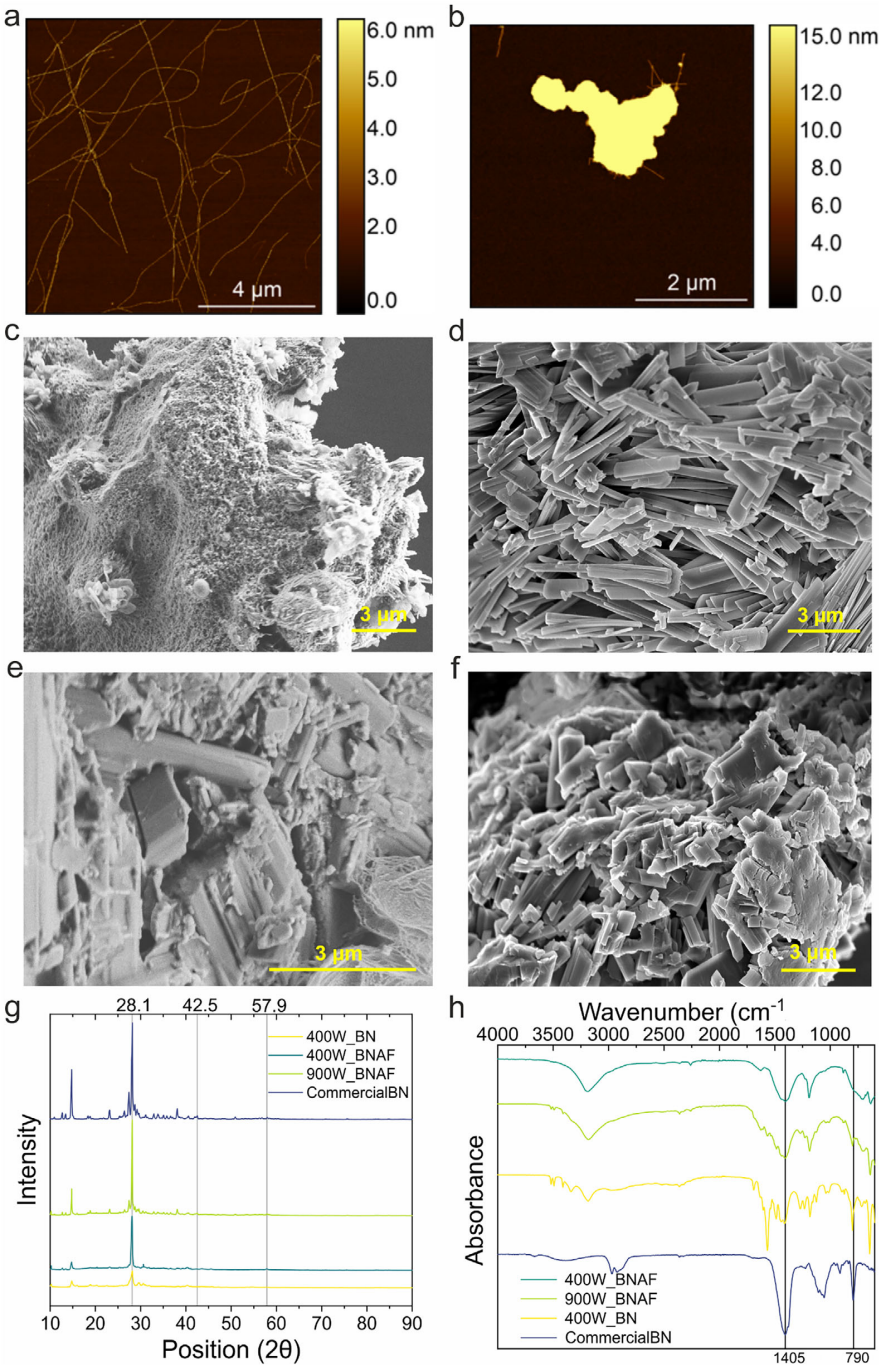
Characterization of BN and BNAF materials. a) AFM image of pure amyloid fibrils showing long, slender morphologies typical of fibrillar protein assemblies. b) AFM image of a BN–amyloid fibril complex, revealing BN aggregates together with fragmented fibrils. c) SEM image of BN processed at 400 W without amyloid fibrils, showing irregular, non‐fibrous morphologies. d) SEM of BN‐rich structures formed at 90 W in the presence of amyloid fibrils, displaying well‐defined whisker‐like structures. e–f) SEM images of BN–amyloid fibril complexes ultrasonicated at 400 and 900 W, respectively, showing dense aggregated structures with embedded BN‐rich fibrous domains. g) XRD patterns comparing high‐power ultrasonicated BN materials with commercial BN, highlighting peak shifts and crystallinity variations. h) FTIR spectra of high‐power ultrasonicated BN materials and commercial BN, showing characteristic B–N bonding vibrations and additional functional groups.

Figure 2a presents atomic force microscopy (AFM) images of pure amyloid fibrils, which appear as long, thin structures characteristic of the typical amyloid fibril morphology. These fibrils display a relatively uniform thickness and length, consistent with previous reports in the literature [40]. In contrast, Figure 2b shows a BN–amyloid fibril complex, in which the fibrils appear significantly shorter, suggesting that high‐power ultrasonication induced fragmentation. The image also reveals BN‐containing aggregates decorated with and partially bridged by amyloid fibrils, indicating close spatial association between the inorganic‐rich domains and the amyloid network. The presence of such aggregates suggests that amyloid fibrils may facilitate the organization and stabilization of BN‐rich hybrid structures. Based on previous reports for protein–BN systems, this association is likely supported by non‐covalent interactions such as hydrogen bonding, π–π interactions, and hydrophobic forces [23], although the exact interfacial bonding environment is not directly resolved here.

Figure 2c–f presents scanning electron microscopy (SEM) micrographs of pure BN and hybrid BNAF samples synthesized under different ultrasonication powers. Figure 2c shows BN processed at 400 W without amyloid fibrils. The surface of such produced BN appears irregular and compact, lacking any discernible fibrous or whisker‐like morphology, indicating that in the absence of amyloid fibrils, BN does not readily form nanofibers under these conditions.

In contrast, Figure 2d depicts BN nanofibers obtained at a low ultrasonication power of 90 W in the presence of amyloid fibrils. Well‐defined, elongated BN whiskers are clearly visible, with lengths and diameters comparable to those reported in literature [41]. However, the fibers here appear less ordered and more randomly oriented compared to the aligned morphologies observed by Zhou et al. [41], likely due to the mechanical agitation from ultrasonication, which induces greater dispersion and reduces alignment compared to mild stirring. This suggests that amyloid fibrils act as scaffold‐mediating agents that favor the formation and organization of elongated BN‐rich structures even at low ultrasonication power. Figures 2e and f show samples ultrasonicated at higher powers (400 W and 900 W, respectively) in the presence of amyloid fibrils. The intended aim of these power settings was to partially exfoliate the BN whiskers into nanosheets via a top‐down peeling effect [8]. However, the resulting morphologies do not display typical nanosheet features. Instead, the SEM images reveal dense, compacted aggregates in which BN nanofibers appear embedded or partially encapsulated within a more consolidated matrix. This densification may be due to the collapse or fusion of fibril–BN assemblies under intense cavitation forces, suggesting that excessive ultrasonication disrupts the open, fibrous network and promotes aggregation rather than exfoliation. Figure S1 compares the pH‐dependent electrophoretic mobility of AF and BNAF. These results, therefore, provide quantitative evidence that the electrokinetic behavior of the fibrils changes upon incorporation of the BN phase, supporting the existence of interfacial interactions between amyloid fibrils and BN‐containing domains. Figure 2g further shows the X‐ray diffraction (XRD) patterns of pure BN (400 W without amyloid fibrils), hybrid BNAF materials (400 W and 900 W), and a commercial BN for reference. The commercial BN sample displays sharp, well‐defined diffraction peaks, characteristic of a highly crystalline layered BN structure [42] with dominant reflections corresponding to the (002), (100), and (110) planes of BN [43]. All synthesized samples, particularly the hybrid BNAF materials, exhibit their most intense peaks at slightly shifted positions when compared with the ideal h‐BN structure, namely 28.1 ° (002), 42.5 ° (100), and 57.9 ° (110). Such peak shifts have been documented for structurally altered BN forms [44], and may indicate changes in interlayer spacing, possible phase modifications, or lattice strain induced by ultrasonication and hybridization with amyloid fibrils.

Both hybrid BNAF samples show only slight peak broadening and moderate intensity variations compared to commercial BN, consistent with a layered BN‐like crystalline structure containing some defects and modestly reduced crystallite size [44, 45]. These effects may arise from strain during synthesis or partial amorphization due to high‐energy processing. In contrast, the 400 W BN sample without amyloid fibrils shows markedly reduced peak intensity and broader reflections, indicative of lower crystallinity and a more amorphous‐like structure [46]. This supports the interpretation that amyloid fibrils assist the formation and structural organization of the BN‐like phase, leading to increased ordering relative to the protein‐free BN control. The crystallinity index (CI), calculated using Equation (1) from the XRD profiles, is summarized in Table 1. Both hybrid samples and the commercial BN show significantly higher CI values than the protein‐free 400 W BN, with the 400 W BNAF material achieving the highest crystallinity (26.2%). These results indicate that the AF–assisted synthesis enhances BN crystal formation and ordering relative to the protein‐free BN sample, while also possibly altering lattice parameters, as reflected in the observed peak shifts. In the following, we therefore refer to a layered BN‐like crystalline phase with increased crystallinity compared to the protein‐free BN control, without implying single‐crystal or defect‐free BN. Literature reports similarly attribute these features to doped or structurally tuned BN systems (e.g., materials with intentionally modified interlayer spacing, stacking order, or defect density) [47].

**TABLE 1:** CI of commercial BN, BN without amyloid fibrils, and hybrid BNAF materials, calculated from XRD data.

BN 400 W	BNAF 400 W	BNAF 900 W	BN Commercial
15.7%	26.2%	20.1%	20.3%

To gain insights into the chemical bonding and molecular structure, Fourier Transform Infrared (FTIR) spectroscopy was performed on the dried samples, with commercial BN serving as the reference (Figure 2h). The spectra of the two hybrid BNAF materials, the commercial BN, and the 400 W BN sample without amyloid fibrils, all display the two characteristic BN peaks: at ∼1405 cm^−1^ corresponding to in‐plane B─N stretching vibrations, and at ∼790 cm^−1^ assigned to out‐of‐plane B–N–B bending vibrations [42]. These peak positions align well with reported BN signatures in the ranges 1320–1450 cm^−1^ and 760–790 cm^−1^ [42, 48].

X‐ray photoelectron spectroscopy (XPS) further supports the incorporation of a boron‐containing inorganic phase into the hybrid material (Figures S2–S4). Compared with AF, the survey spectrum of BNAF_400 W shows the appearance of a B signal together with an increased N/C ratio. In the high‐resolution spectra, the C 1s and O 1s regions remain largely dominated by the amyloid/protein framework, whereas the N 1s spectrum of BNAF_400 W exhibits an additional lower‐binding‐energy contribution relative to AF, indicating a modified nitrogen environment in the presence of the BN‐derived phase. The B 1s region of BNAF_400 W can be deconvoluted into components consistent with a BN‐derived phase containing oxygenated surface boron environments, likely reflecting surface oxidation and/or modified interfacial chemistry in the hybrid material. Overall, these XPS data support the presence of a BN‐derived boron‐containing phase with modified surface/interfacial chemistry in the hybrid material.

The commercial BN sample exhibits sharp, well‐defined peaks, indicative of high purity and structural order. The hybrid BNAF samples closely resemble this spectral profile, with both characteristic BN bands clearly visible along with additional peaks near ∼1060 cm^−1^ and ∼600 cm^−1^, consistent with B─N─O‐ and B─O‐type environments commonly reported for oxygen‐containing BN and BN‐based hybrids [23, 42]. In contrast, the 400 W BN sample without amyloid fibrils shows a noisier spectrum with broadened peaks, suggesting polydispersity, incomplete BN formation, and the presence of unreacted precursors.

A broad feature between 3200–3400 cm^−1^ is observed in all samples, corresponding to N–H and O–H stretching vibrations, indicative of residual hydroxyl groups or adsorbed moisture. Taken together, the FTIR results support the formation of BN‐like bonding environments in the hybrid samples under mild processing conditions, without the need for temperatures above 1000°C or high‐pressure processes typical of conventional BN production [10, 22].

**FIGURE 3:**
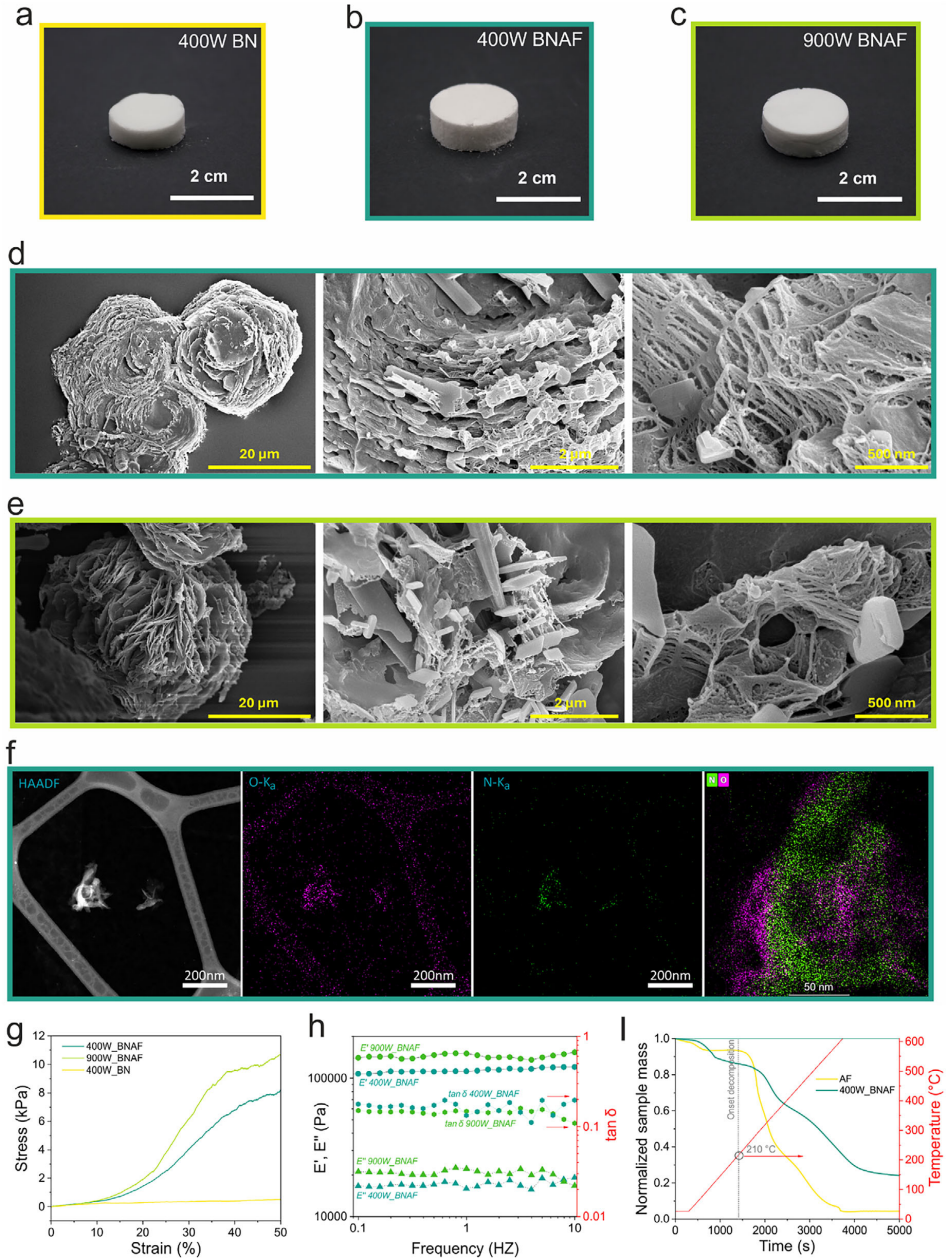
Characterization of BN and WPI amyloid fibril (BNAF) aerogels. a) Aerogel produced at 400 W ultrasonication without amyloid fibrils. b) 400 W hybrid BN–amyloid fibril aerogel. c) 900 W hybrid BN–amyloid fibril aerogel. d) Multi‐magnification SEM images of the 400 W BNAF aerogel fracture surface (scale bars: 20, 2, and 500 nm, from left to right). e) Multi‐magnification SEM images of the 900 W BNAF aerogel fracture surface (scale bars: 20, 2, and 500 nm, from left to right). f) STEM–EDS characterization of the 400 W BN–amyloid hybrid, providing local compositional support for nitrogen‐containing inorganic domains. HAADF image, O‐K and N‐K elemental maps, and drift‐corrected overlay map. g) Compression tests at 50% strain comparing the three aerogels. h) DMA showing E′, E″, and tan δ of aerogels across the frequency range sampled. i) TGA of pure amyloid fibrils and the 400 W hybrid BN–amyloid fibril aerogels.

Figure 3a–c shows the visual appearance of aerogels obtained by freeze‐drying BN and BNAF samples immediately after ultrasonication. The aerogel prepared at 400 W without amyloid fibrils (Figure 3a) appears thinner, with a finer and more fragile microstructure compared to those reinforced with WPI amyloid fibrils. Among the protein‐reinforced aerogels, the 400 W sample (Figure 3b) is noticeably thicker than the 900 W sample (Figure 3c), suggesting that higher ultrasonication power promotes greater shrinkage or structural collapse during processing. This is likely due to increased fibril fragmentation at higher power, which negatively impacts aerogel thickness [49].

To directly visualize the internal architecture of the hybrid aerogels, we performed SEM imaging on fracture surfaces of the freeze‐dried BNAF monoliths at multiple magnifications (Figure 3d, e). At low magnification, both samples exhibit a porous, hierarchical framework composed of micron‐sized aggregates of BN–fibril hybrids. The lower‐magnification SEM images (Figure S5) further demonstrate that these features are not merely local fracture‐surface artifacts, but extend over hundreds of micrometers as a continuous macroporous skeleton with visible interdomain connectivity. The 400 W BNAF sample exhibits more compact, “onion/rosette‐like” agglomerates with pronounced lamellar textures, indicative of partial restacking and dense packing of BN‐rich domains (Figure 3d, left). In contrast, the 900 W BNAF sample shows a more open and fibrillar, web‐like morphology, with abundant filamentous features protruding from the aggregates and bridging neighboring domains (Figure 3e, left), consistent with improved dispersion and network formation at higher ultrasonication power. At intermediate magnification (2 µm scale), both aerogels reveal a continuous scaffold composed of BN platelet‐like structures integrated within an organic matrix, but the 900 W sample displays more uniformly distributed structural elements and fewer thick, stacked regions, whereas the 400 W sample contains more extended lamellar/stacked features (Figures 3d,e, middle). High‐magnification images (500 nm scale) further resolve a nanofibrillar network spanning micron‐scale voids, with BN platelets embedded/anchored within the fibrillar skeleton (Figures 3d, e, right). The SEM observations suggest that increasing ultrasonication power promotes a more connected hybrid framework, providing a plausible microstructural basis for the higher stiffness and compressive strength of the 900 W BNAF aerogel observed in mechanical testing.

To support the SEM observations with a bulk porosity measurement, mercury intrusion porosimetry was performed to evaluate the intrudable pore volume and accessible porosity of the aerogel networks. The results show high Hg‐accessible porosity for all aerogels, with AF exhibiting ∼90% and both hybrid BNAF aerogels ∼87%. The slightly lower porosity of the hybrids is consistent with their higher inorganic BN‐rich solid fraction and modest densification of the load‐bearing skeleton upon hybrid formation. Importantly, the similarly high porosity of the 400 and 900 W BNAF samples confirms that both hybrid aerogels possess an open, accessible porous network. The comparable total porosity further indicates that ultrasonication primarily tunes pore architecture and connectivity rather than the overall void fraction, in agreement with the SEM observations.

To further complement the SEM and Hg‐porosimetry results, the pore structure of the hybrid BNAF and pure AF aerogels was evaluated by N_2_ adsorption–desorption at 77 K. All samples show relatively low BET surface areas (S_BET_ < 5 m^2^ g^−^^1^), consistent with an aerogel architecture dominated by large, micron‐scale macropores that are not fully accessible to N_2_ sorption. Nevertheless, the incorporation of BN increases both the accessible surface area and pore volume compared to AF, with S_BET_ rising from 2.91 m^2^ g^−^^1^ for AF to 3.41 and 4.43 m^2^ g^−^^1^ for BNAF prepared at 400 W and 900 W, respectively (Table 2). Similarly, the total pore volume increases from 0.0039 cm^3^ g^−^^1^ (AF) to 0.0128–0.0136 cm^3^ g^−^^1^ for the BNAF aerogels, indicating a larger fraction of N_2_‐accessible mesoporosity in the hybrids. The adsorption–desorption isotherms exhibit a pronounced hysteresis loop and a steep uptake at high relative pressure, which is characteristic of mesoporous materials with capillary condensation and interparticle voids, consistent with mesopores arising from interstitial voids between aggregated BN domains and the fibrillar scaffold. In agreement, DFT pore‐size analysis reveals that AF contains primarily small mesopores with characteristic pore widths around ∼5–6 nm, whereas both BNAF aerogels display a dominant mesopore population centered at ∼29 nm (Table 2). The 900 W sample also shows an additional smaller‐pore contribution (∼4.9 nm), suggesting that higher ultrasonication power can introduce finer mesoporous features, likely due to stronger fragmentation and reorganization of the hybrid building blocks.

**TABLE 2:** BET and pore‐size distribution results.

	S_BET_ (m^2^/g)	V_p_ (cm^3^/g)	Mode Pore size (nm)
AF	2.9	0.005	5‐6
400 W BNAF	3.4	0.017	29
900 W BNAF	4.4	0.019	29

To further probe the local composition of the hybrid BN‐containing materials, scanning transmission electron microscopy‐energy dispersive X‐ray spectroscopy (STEM–EDS) analysis was performed on the 400 W sample (Figure 3f; Figure S6). Representative data (Figure 3f) show flake‐like particles in the high‐angle annular dark field (HAADF) image and a clear spatial separation between the O─K and N─K elemental maps. In particular, localized N‐rich domains are observed that do not coincide with the oxygen‐rich background/support, and the corresponding EDS spectrum shows a pronounced N signal. Because boron could not be directly mapped in the present EDS configuration, the analysis was based on identifying regions with a strong N signal spatially distinct from the C/O background. While nitrogen mapping alone cannot unambiguously prove BN formation, these STEM–EDS observations provide local compositional support that is consistent with the XRD, FTIR and XPS evidence for a BN‐rich phase within the hybrid material. The STEM–EDS observations are therefore consistent with the presence of nitrogen‐containing inorganic domains embedded in the amyloid‐supported hybrid matrix, while more definitive identification of local bonding environments would require advanced techniques such as EELS, or optimized boron‐sensitive elemental mapping.

Compression testing revealed a pronounced mechanical enhancement in the amyloid fibril–reinforced aerogels compared to the protein‐free control (Figure 3g). At 50% strain, the 900 W hybrid aerogel reached ∼9 kPa, and the 400 W hybrid reached ∼7 kPa before brittle fracture, whereas the non‐reinforced BN aerogel did not exceed 1 kPa. These results demonstrate the significant reinforcing effect of amyloid fibrils. Comparable BN‐based aerogels have been reported in the literature, such as BN nanotube aerogels reinforced with reduced graphene oxide (rGO) by Wang et al., which achieved a maximum stress of ∼3.5 kPa [50], lower than the amyloid fibril–reinforced samples here. However, unlike the rGO‐reinforced aerogels, which exhibited compression resilience and recovery, the BN–amyloid fibril aerogels in this work did not recover their original shape once the load was released.

Dynamic mechanical analysis (DMA) was performed to further evaluate the mechanical properties of the aerogels (Figure 3h). Both BNAF samples exhibit viscoelastic solid behavior, with storage modulus (E′) consistently higher than loss modulus (E″) across the frequency range sampled. The Aerogel prepared using 900 W ultrasonication showed significantly higher E′ and E″ values, indicating a higher stiffness and mechanical reinforcement due to improved dispersion and network integration of BN‐rich domains. The loss tangent (tan δ) remained below 0.2 for both samples, confirming elastic‐dominant behavior. The slightly lower tan δ values of the 900 W aerogel suggest a more robust network with reduced energy dissipation. Overall, these results demonstrate that increasing ultrasonication power enhances the stiffness and solid‐like character of BNAF aerogels.

Thermogravimetric analysis (TGA) was performed to assess the thermal stability and composition of the aerogels (Figure 3i). The 400 W BN–amyloid fibril aerogel retained ∼27% of its mass after heating to 900°C in air, indicating the presence of thermally stable BN as well as residual components. The mass loss between 90°C–370°C can be attributed to the degradation of residual organic components, including the protein fraction and melamine‐derived intermediates [51]. A sharper decrease between 370–410°C is associated with the decomposition of melamine–boric acid condensation products and further release of volatile species [52]. The stable residue above 900°C likely also contains contributions from unreacted boric acid, which typically shows only ∼40%–50% weight loss under similar conditions [53], as well as carbonaceous remnants of the protein fraction, which begins decomposing at ∼210°C [23]. We emphasize that the TGA experiment is used here to assess composition and thermal decomposition behavior, rather than to claim preservation of the aerogel architecture at high temperature. Because the amyloid fibrils form a major part of the load‐bearing scaffold, substantial protein degradation is expected to compromise the original porous architecture. Therefore, the present hybrid aerogels are intended for moderate‐temperature acoustic applications rather than extreme high‐temperature use.

### 2.2 Sound Insulation

BN aerogels are promising candidates for acoustic shielding due to their low density, high porosity, and strong sound attenuation capabilities [9]. Their lightweight, interconnected network structure enables both absorption and dissipation of sound energy, making them suitable for noise reduction applications.

**FIGURE 4:**
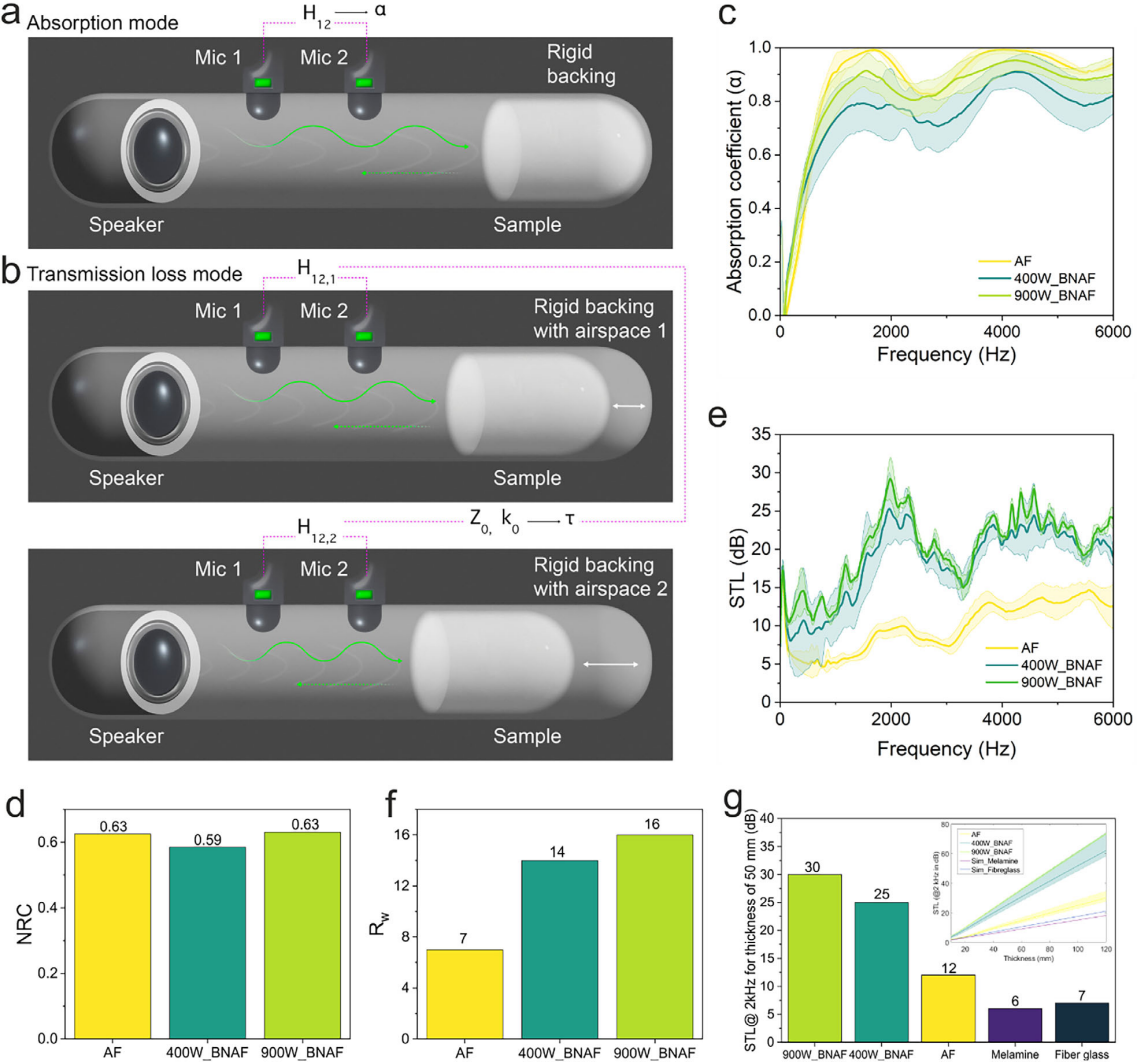
Acoustic performance of BN–amyloid fibril aerogels. a) Impedance tube setup for sound absorption measurements according to ISO 10534‐2. b) Setup for normal sound transmission loss measurements based on the 2M2L method. c) α spectra of pure amyloid fibrils and hybrid BN–amyloid fibril aerogels. d) *NRC* values. e) *STL* spectra. (f) *R_w_* values. (g) *STL* at 2 kHz and for a 50 mm thickness (inset: Simulated *STL* as a function of thickness), for the three aerogels compared with reference materials from the literature [61].

The sound‐insulating performance of the aerogels (thickness (*t*) of 40–45 mm) was evaluated using two methods: sound absorption and sound transmission loss, both measured with an impedance tube setup. Densities were 0.047 g/cm^3^ for the amyloid fibril aerogel and 0.096 g/cm^3^ for the hybrids. For absorption measurements, the ISO 10534‐2 [54] two‐microphone method was employed (Figure 4a). Here, the sound absorption coefficient is determined at a given frequency from a superposed normal incident sound wave and the reflected sound wave from the sample mounted with a rigid backing; the unreflected portion is absorbed. For transmission loss measurements, the 2M2L method was used with two microphones and two different plenum depths [55] (airspace lengths) (Figure 4b). This configuration allows for quantification of the bulk acoustic properties of the porous material, namely the characteristic wave impedance Z_0_, and the wavenumber k_0_ of sound travelling in the sample.

The sound absorption coefficients (α) of the two hybrid BN–amyloid fibril aerogels (400 and 900 W) and the pure amyloid fibril aerogel are shown in Figure 4c. All samples exhibit α > 0.8 across a wide frequency range, indicating excellent sound absorption. Although pure amyloid fibrils have a slightly higher α, the hybrid aerogels still demonstrate strong absorption despite partial fibril fragmentation during processing. The higher ultrasonication power (900 W) resulted in slightly improved absorption compared to the 400 W hybrid, despite the two hybrids having similar total Hg‐accessible porosity. This difference is therefore attributed primarily to pore architecture and connectivity rather than total porosity alone. Noise reduction coefficients (NRC) confirm this trend, with the 900 W hybrid and pure amyloid samples reaching 0.63, and the 400 W hybrid reaching 0.59 (Figure 4d). The lower NRC of the 400 W BNAF sample is consistent with its more compact/lamellar and locally stacked morphology observed by SEM, which can reduce effective acoustic coupling into the porous network. In contrast, the 900 W BNAF aerogel exhibits a more open, web‐like framework and higher N_2_‐accessible surface area, favoring more efficient viscous and thermal dissipation of incident sound. These *NRC* values are comparable to elastic ceramic nanofibrous aerogels (*NRC* ═ 0.59) reported by Cao et al., [56].

Due to the porous nature of aerogels, sound can pass through easily; therefore, normal sound transmission loss (*STL*) was determined from the measured bulk acoustic properties [36] (Figure 4e). Both hybrid BN aerogels showed substantially higher STL than pure amyloid fibrils, averaging ∼20–25 dB across the frequency range compared to ∼10 dB for the control. This comparison directly highlights the role of BN in the hybrid aerogels: while the amyloid fibril network already provides an efficient porous absorber, incorporation of BN‐rich domains increases the effective solid fraction, stiffness, and dynamic impedance of the porous layer, thereby improving sound shielding and STL while maintaining high absorption. The 900 W hybrid showed superior STL performance, consistent with a higher effective acoustic impedance and stronger reflection. For applications requiring higher sound insulation, adding airtight surface layers could further enhance performance by increasing acoustic impedance and reflection [57]. From a mechanistic viewpoint, the 2M2L analysis allows us to interpret the acoustic response in terms of the characteristic impedance *Z*_0_ and complex wavenumber *k*_0_ of the porous medium, which embody the combined effects of sound propagation in the fluid phase described by flow resistivity, tortuosity, porosity, and viscous/thermal losses as well as in elastic wave propagation via the solid skeleton of the absorber. Mercury intrusion porosimetry indicates similarly high Hg‐accessible (intrudable) porosity for both hybrid aerogels (∼87%), suggesting that ultrasonication primarily tunes pore architecture and connectivity rather than the overall void fraction. Consistent with this, SEM reveals distinct organization of the BN–fibril assemblies: the 400 W aerogel contains more compact/lamellar, locally stacked domains, whereas the 900 W aerogel exhibits a more web‐like, bridged framework with more uniformly distributed junctions. Such differences are expected to alter effective flow pathways, enhance the coupling between the fluid and solid domain and affect impedance matching. In qualitative terms, a microstructure with better impedance matching to air favors broadband absorption, whereas a framework with higher effective impedance and coupling between the solid and fluid phase promotes stronger reflection and hence higher STL, potentially at the expense of absorption at certain frequencies. In both 400 W and 900 W cases, the large internal surface area and interconnected pore network enable viscous and thermal dissipation of acoustic energy, which is most effective at mid–high frequencies. In addition, the stronger fluid–frame coupling, including frame motion and inertial coupling, increases the effective dynamic impedance of the porous layer and tends to increase impedance mismatch with air, thereby enhancing reflection and boosting STL.

As shown in Figure S7, applying the Jaouen et al. inversion to the AF samples yields open‐porosity values close to 0.9 with acceptable uncertainty, in good agreement with Hg porosimetry, and provides a plausible order of magnitude for the flow resistivity. In contrast, for the hybrid BNAF aerogels, particularly the 400 W samples, the frequency‐dependent JCAL inversion produces large scatter and ill‐conditioned/nonphysical estimates, which prevents reliable determination of the remaining model parameters that require porosity and flow resistivity as inputs. This behavior is consistent with known limitations of impedance‐tube‐based JCAL identification when the measured dynamic bulk modulus and mass density are influenced by frame‐related effects, effectively violating the rigid‐/limp‐frame assumptions underlying the inversion and leading to ill‐conditioned parameter estimates. We therefore refrain from reporting a fully parameterized JCAL dataset for the hybrid aerogels and instead rely on the robust bulk descriptors *Z*_0_ and *k*_0_ together with porosity and microstructural characterization to interpret the absorption/STL trends. The ensued hybrid BN–amyloid fibril aerogels combine high sound absorption with strong transmission loss, reaching values that are competitive with representative reported aerogel‐based acoustic materials. For example, hybrid silica–cotton aerogels (*t* of 9 mm) achieved α up to 0.89 [58], and silica aerogels with additives (*t* of 20 mm) showed α ═ 0.84 and *STL* ≈ 9.3 dB [59]. A broader comparison with representative aerogel‐based acoustic materials from the literature is provided in Table S1. The hybrids in this work achieve comparable absorption and significantly higher *STL* values.

To place these results in a practical context, we also compare with materials commonly used in acoustic insulation. Weighted sound reduction index (*R_w_*) values (Figure 4f) were 14–16 dB for the hybrid materials, compared to 7 dB for the pure amyloid sample. These values surpass those of PVC and bitumen layers, and are close to resilient rubber‐based layers (*R_w_* ═ 18 dB) [60]. To rightfully compare our results in the context of available literature, we evaluated the *STL* as a function of thickness (Figure 4g, inset) and benchmarked our materials against two conventional porous insulators. As observed, at 2 kHz and for a 50 mm thickness, the 900 W hybrid (ρ ═ 0.096 g/cm^3^) achieved an *STL* of 30 dB, far outperforming melamine foam (∼6 dB, ρ ═ 0.0084 g/cm^3^) [61] and fiberglass (∼7 dB, ρ ═ 0.0055 g/cm^3^) [61] under comparable conditions (Figure 4g).

This combination of high sound absorption and transmission loss in a single, monolithic material is particularly noteworthy for lightweight, moderate‐temperature acoustic applications, where balancing absorption, shielding, and low density remains challenging [62]. The hybrid BN–amyloid fibril aerogels achieve this performance while remaining extremely lightweight compared to conventional polymeric or mineral‐based solutions, offering advantages in applications where weight and thickness are constrained [63]. Beyond conventional soundproofing panels, such materials could be considered for lightweight interior partitions, device enclosures, or packaging applications where moderate‐temperature operation, low density, vibration damping, and sound attenuation are desirable. Their tunable network structure also opens opportunities for further performance optimization, for example, by incorporating surface skins or multilayer assemblies to further boost *STL* for more demanding use cases [36].

## 3 Conclusions

In this work, we have developed BNAF aerogels through a bottom‐up ultrasonication and freeze‐drying process using whey protein amyloid fibrils as inexpensive renewable scaffolds. The incorporation of amyloid fibrils significantly improved BN crystallinity, mechanical reinforcement, and aerogel formation compared to protein‐free BN. Structural analysis supports successful hybridization, with fibrils assisting the formation, dispersion, and organization of BN‐rich domains under moderate processing conditions. The resulting aerogels displayed low density and improved mechanical integrity relative to the protein‐free control, together with thermal behavior consistent with the presence of a BN‐rich inorganic residue. Most importantly, these hybrid aerogels showed strong acoustic performance, with sound absorption coefficients above 0.8 and STL of 30 dB at a thickness of 50 mm, comparing favorably with common lightweight acoustic materials. Their high shielding efficiency per density highlights their promise for moderate‐temperature, weight‐sensitive noise‐reduction applications.

## 4 Experimental Section/Methods

### 4.1 Materials

Whey protein isolate (WPI) was purchased from Fonterra, New Zealand. Boric acid (ACS reagent, ≥ 99.5%), melamine (99%), and hexagonal boron nitride ink were purchased from Sigma‐Aldrich, USA.

### 4.2 Preparation of the Hybrid Amyloid Fibril BN Solution

A 4 wt.% WPI solution was prepared by dissolving 10 g of WPI powder in 240 mL of Milli‐Q water under continuous stirring at room temperature until no visible agglomerates remained. Subsequently, 10 g of boric acid was added and fully dissolved. The pH of the solution, which decreased to 3.6 upon boric acid addition, was adjusted to 2.0 using 2 m HCl. The mixture was then heated at 90°C in an oil bath under continuous stirring for 5 h (Figure 1). After heating, the solution was cooled to 4°C. This thermal treatment at 90°C promotes the conversion of WPI monomers into amyloid fibrils and the formation of boric acid–fibril complexes. The absence of boric acid re‐sedimentation or crystallization after cooling, phenomena typically observed without protein incorporation, supports the occurrence of such complexation.

To prepare the BN–amyloid fibril hybrid suspension, 5 g of melamine was added to 100 mL of the amyloid fibril–boric acid WPI solution. The mixture was ultrasonicated for 5 min using a Compact Ultrasonic Laboratory Device UP100H (Hielscher Ultrasonics GmbH, Teltow, Germany) equipped with an MS2 sonotrode. Ultrasonication was performed at 90 W in continuous mode, yielding a homogeneous white suspension of the hybrid BN–amyloid fibril material (BNAF complex).

### 4.3 Preparation of Hybrid BN‐Amyloid Fibrils Aerogels

Hybrid BN–amyloid fibril aerogels were produced through an additional ultrasonication step. The 100 mL homogeneous nanofiber suspension was ultrasonicated for 20 min at either 400 or 900 W using an Ultrasonic Homogenizer (Scientz Biology, Zhejiang, China). Following ultrasonication, 2 mL aliquots of the white suspension were immediately transferred into aluminum molds and frozen at −80°C. The frozen samples were then freeze‐dried for 48 h using a FreeZone Plus 4.5 freeze dryer (Labconco, United States).

### 4.4 Characterizations

For FTIR, the ultrasonicated suspension was dried at 40°C for 48 h, and the resulting BN powder was analyzed on a Vertex 70 spectrometer (Bruker, USA) over 4500–500 cm^−1^. A small sample (∼straw tip) was placed on the detector, with spectra recorded in triplicate and averaged.

The electrophoretic mobility of AF and Ch was measured by using a Malvern Nano‐Zetasizer. For XRD, BN powder from the same drying process was analyzed on a PANalaytical Empyrean instrument using Cu Kα radiation (λ ═ 1.5418 Å, 45 mA, 40 kV). Measurements were made in the 2θ range of 10–90° with a step size of 0.016° and a total acquisition time of 1 h. The crystallinity index (*CI*) [64] was calculated as the ratio of the crystalline peak area (*A*_a_) to the total area of crystalline and amorphous peaks, using XRD data Equation (1):

(1) $$CI=\frac{A_{c}}{A_{c}+A_{a}}\;\times 100$$

where *A*_c_ is the area under the crystalline peaks, *A*_a_ is the area under the amorphous peaks, and *CI* is expressed as a percentage.

SEM was performed using a TFS Magellan 400 (Thermo Fisher Scientific Inc., Massachusetts, USA). Samples were sputter‐coated with a 5 nm Pt/Pd (80:20) layer to minimize charging during imaging.

XPS analysis was performed using an AXIS Supra spectrometer (Kratos Analytical, UK) equipped with a hemispherical analyser and a monochromatic Al K‐α source (1487 eV) operated at 15 mA and 15 kV. The XPS spectra were collected from an analysis area of 700 × 300 µm^2^ at a take‐off angle of 90°. Pass energies of 160 eV and 20 eV were used for survey and high‐resolution scans, respectively. A 3.1 V bias was applied to the sample to prevent charge build up on the sample surface. The binding energies (BEs) were charge‐corrected based on the C 1s of adventitious carbon at 284.8 eV. The data processing was performed using the ESCApe software. The XPS samples were by prepared by pressing the powdered particles on carbon tape.

AFM measurements were conducted under normal atmospheric conditions at RT, with no heating of the sample. Samples were diluted to 0.0001 wt%, and 100 µL were pipetted onto mica. After 10 min, the mica was rinsed with MiliQ water and dried with compressed air. AFM images were acquired using a Bruker Icon 3 (Bruker, Massachusetts, USA) in soft‐tapping mode with an RTESPA‐150 probe operating at 150 kHz.

N_2_ physisorption was conducted at −196°C using an Autosorb 6300 instrument (Anton Paar), covering the p/p0 range from 0.005 to 0.99. Samples were degassed at 120°C for 3 h under vacuum. Surface area and pore volume were obtained using the Brunauer‐Emmett‐Teller (BET) and Barrett‐Joyner‐Halenda (BJH) models, respectively.

Hg intrusion porosimetry was performed using an Autopore IV 9500 instrument (Micromeritics) with pressures ranging from 0.5 to 30,000 psi. The porosity of the samples was calculated from the apparent and bulk density (determined at 0.5 psi) measurements.

The morphology and local elemental distribution of the BNAF hybrid materials were analyzed by STEM using a Talos F200X (Thermo Fisher Scientific, USA) operated at 200 kV and equipped with a SuperX EDS system with four silicon‐drift detectors. HAADF, low‐angle annular dark‐field (LAADF), and bright‐field (BF) STEM signals were collected simultaneously. TEM specimens were prepared by drop‐casting dilute dispersions onto Cu grids coated with lacey‐carbon‐supported graphene films; the support films were glow‐discharged prior to deposition to improve flake dispersion. Elemental maps were extracted from EDS spectrum‐image datasets by monitoring the spatial distribution of N and O signals.

Compression tests were performed using a ZwickRoell Z010/TH2 (ZwickRoell, Ulm, Germany) in plate–plate geometry. BN aerogels were tested under stresses of 0.1%–10 kPa and strains of 0%–50%, with the upper plate lowered at a constant speed of 5 µm/s while recording force.

Dynamic mechanical analysis (DMA) of the aerogels was carried out in tensile mode using a TA Instruments RSA3 after conditioning at 23°C and 48% relative humidity for 4 days. The tests were performed with a gauge length of 17 mm under a preload of 0.05 N. A frequency sweep (compression) was conducted, beginning from 0.05 to 100 Hz.

TGA was conducted on a TGA/DSC3+ instrument (Mettler Toledo, Greifensee, Switzerland) to assess the thermal stability of the aerogels. Samples were heated from 25°C to 900°C under a nitrogen atmosphere, and the mass change was recorded as a function of time and temperature.

### 4.5 Acoustic Impedance Tube Measurements

Acoustic performance was evaluated using an impedance tube with a two‐microphone method for absorption and transmission loss measurements from samples (∼40 mm height, 26 mm diameter), which included three freeze‐dried aerogels: BN–amyloid fibrils prepared at 400 and 900 W, and pure amyloid fibrils. Each was mounted in a 26 mm custom sample holder with a rigid backing, and a broadband sound signal was generated by a loudspeaker. The reflection coefficient was measured, and the average absorption coefficient was calculated from three replicates per sample type. The impedance tube was Type 4206 equipped with Type 4598 A ¼′‐microphones (both Brüel and Kjaer, Denmark). Signals were generated and measured with a 4‐channel data acquisition system, Type USB 4431 from National Instruments, Austin, US. Data acquisition and signal processing were done with the Software Tubecell from Matelys Research Labs, Vaulx‐en‐Velin, France. As an output signal for excitation of the impedance tube, 8 pseudo‐random white noise samples were generated, and each of them was repeated four times. The sound pressure was sampled with the microphones simultaneously, and an FFT analysis with 1 Hz frequency resolution was conducted, resulting in 32 transfer functions that were averaged for each measurement. The microphone spacing was 50 mm, which, in combination with the 30 mm tube, allows for a measurement range from 200 to 6400 Hz.

### 4.6 Sound Absorption

The normal sound absorption coefficient was determined according to the procedure described in ISO 10534‐2 [54]. The reflection coefficient r, the absorption coefficient α, as well as the normalized surface impedance *Z_sn_* were determined using the Tubecell software from the transfer function between two upstream microphone positions, *Ĥ_12_ ═ p_2_/p_1_*, which were corrected for a possible phase lag between the microphone signals.

### 4.7 Sound Transmission Loss

The acoustic bulk properties of a porous material, namely the characteristic wavenumber *k_0_* and the characteristic wave impedance *Z_0_*, were determined using the 2‐microphone 2‐load (2M2L) method [55, 65].

This method is based on two sound absorption measurements that are carried out according to ISO 10534‐2 [54] on the same porous material sample, but mounted with two different airspaces between the back surface of the sample and the rigid termination of the tube. For the investigated samples, measurements were performed with air space depths *L*  ═  55 mm and *L'*  ═  78 mm behind the sample. With the knowledge of the normalized surface impedance *Z_sn_* and *Z'_sn_* from the two measurements and the corresponding airspace depths *L* and *L'* respectively, a set of linear equations can be solved to determine the characteristic wavenumber *k_0_* and the characteristic wave impedance *Z_0_*. These properties describe acoustic wave propagation in the porous medium.

The transfer matrix *T* that describes normal sound propagation from air through a porous material of thickness *h* into air is given by Equation (2) [66]:

(2) $$T=\left[\begin{array}{cc} T_{11} & T_{12}\\ T_{21} & T_{22} \end{array}\right]=\left[\begin{array}{cc} cos\left(k_{0}h\right) & jZ_{0}sin\left(k_{0}h\right)\\ j\frac{1}{Z_{0}}sin\left(k_{0}h\right) & cos\left(k_{0}h\right) \end{array}\right]\;$$

The sound power transmission coefficient *τ* for normal incident sound waves is then given by Equation (3):

(3) $$\tau =\frac{4}{\left|T_{11}+\frac{1}{Z_{air}}T_{12}+Z_{air}T_{21}+T_{22}\right|}$$

With acoustic wave impedance Z_air_ ≈ 413 (Pa s/m) at 20°C and normal conditions. The sound transmission loss (*STL*) is finally given by Equation (4).

(4) $$STL=10\cdot \mathrm{lg}\left(\frac{1}{\tau }\right)$$

The codes to calculate the *STL* for the samples of thickness 50 mm and as well as to show the effect of the sample thickness on the *STL* at 2 kHz were implemented in Matlab.

### 4.8 Single Number Ratings

For a quick comparison of the acoustic performance of materials, single‐number ratings are determined from the frequency spectra. Hereby, first, the FFT‐spectra of α and *STL* were digitally bandfiltered to obtain 1/3‐octave band spectra [67].

For sound absorption, the noise reduction coefficient (*NRC*) was determined from third octave band data according to ASTM C423, taking into account the 250 to 2000 Hz octave band [68].

For sound insulation, the weighted sound reduction index (*R_W_*) was determined in accordance with ISO 717‐1 [69].

## 5 Conclusions

In this work, we developed BNAF aerogels through a bottom‐up ultrasonication and freeze‐drying process using whey protein amyloid fibrils as structural templates. The incorporation of amyloid fibrils significantly improved BN crystallinity, mechanical reinforcement, and aerogel formation compared to protein‐free BN. Structural analyses confirmed successful hybridization, with fibrils facilitating BN nucleation, fiber growth, and enhanced ordering under moderate synthesis conditions. The resulting aerogels displayed low density and good mechanical and thermal stability, making them lightweight yet robust against stress and heat. They also showed excellent sound‐insulating performance, with absorption coefficients above 0.8 and sound transmission loss of 25–30 dB, outperforming many conventional lightweight acoustic materials. Their high shielding efficiency per density highlights their promise in weight‐sensitive sectors such as aerospace and transport.

## Author Contributions

M.P. and R.M. conceptualized the project, supervised the research, and designed studies; M.P. and A.L. performed the experiments from nanobuilding block preparation to hybrid materials fabrication and characterization; S.S. carried out the acoustic measurement and analysis; F.D. carried out TGA, N_2_ physisorption, and XRD measurements; E.B. carried out the AFM and DMA; all authors contributed to evaluating data and writing the final version.

## Conflicts of Interest

The authors declare no conflicts of interest.

## Supporting information




**Supporting File**: adma74088‐sup‐0001‐SuppMat.docx.

## Data Availability

All data supporting the findings of this study are provided in the article and its Supplementary Information. Additional information is available from the corresponding author upon reasonable request.
