# Supplementary material for: Hybrid Amyloid–Boron Nitride Aerogels for Lightweight Sound Insulation
Source: Adv Mater. 2026 Jul 17;38(47):e74088. doi: 10.1002/adma.74088 (PMC13496049; doi:10.1002/adma.74088)
Supplement: Supplementary file 1 [file ADMA-38-e74088-s001.docx]

Supporting Information

**Hybrid Amyloid–Boron Nitride Aerogels for Lightweight Sound Insulation**

Mohammad Peydayesh^1*^, Andrin Lustgarten^1^, Stefan Schoenwald^2^, Enrico Boschi^2,3^, My Hanh Bui^4^, Ali Miserez^4,5^, Felix Donat^6^, Christoph R Müller^6^, Raffaele Mezzenga^1,7*^

*^1^ ETH Zurich, Department of Health Sciences and Technology, 8092 Zurich, Switzerland*

*^2^ Swiss Federal Laboratories for Materials Science and Technology (Empa), Ueberlandstrasse 129, Duebendorf, 8600, Zurich, Switzerland*

*^3^University of Copenhagen, Plant Glycobiology, Thorvaldsensvej 40, 1871 Frederiksberg C, Denmark*

*^4^Centre for Sustainable Materials (SusMat), School of Materials Science and Engineering, Nanyang Technological University, Singapore, Singapore.*

*^5^School of Biological Sciences, Nanyang Technological University, Singapore, Singapore.*

*^6^ Department of Mechanical and Process Engineering, ETH Zürich, Leonhardstrasse 21, CH-8092 Zürich, Switzerland*

*^7^ ETH Zurich, Department of Materials, 8093 Zurich, Switzerland*

**Figure S1.** Electrophoretic mobility of AF and the BNAF.

**Figure S2.** XPS survey spectra of AF and BNAF_400W. The hybrid sample shows the appearance of boron together with an increased N/C ratio relative to AF, consistent with incorporation of a boron-containing inorganic phase into the amyloid matrix. The overlap region of B 1s and Cl 2p is indicated.

**Figure S3.** High-resolution XPS spectra of the N 1s and B 1s regions for AF and BNAF_400W. Compared with AF, BNAF_400W exhibits an additional lower-binding-energy contribution in the N 1s region, consistent with a modified nitrogen environment. The B 1s region of the hybrid can be fitted with components more consistent with oxygen-containing boron environments than with ideal stoichiometric BN.

**Figure S4.** High-resolution XPS spectra of the C 1s and O 1s regions for AF and BNAF_400W. The C 1s region remains dominated by the protein framework, whereas the O 1s region of the hybrid shows enhanced oxygen-containing contributions, consistent with the presence of oxygenated boron environments and modified interfacial chemistry.

**Figure S5.** Low-magnification SEM images of the 400 W and 900 W BNAF aerogels. Images are shown at scale bars of 200 µm and 500 µm to visualize the larger-scale aerogel skeleton and macroporous connectivity. Both aerogels display an open porous architecture over hundreds of micrometers. The 400 W BNAF aerogel shows larger lamellar/flocculated aggregates and open macroporous regions, whereas the 900 W BNAF aerogel shows a more uniformly distributed porous network with smaller and more dispersed structural elements.

**Figure S6.** EDS spectrum of the 400 W BN–amyloid hybrid.

**Figure S7.** Inverse identification of selected JCA-type parameters from impedance-tube measurements following the analytical procedure of Jaouen et al.^[1]^ Frequency-dependent estimates of open porosity (left panel) and airflow resistivity (right panel) for AF and hybrid BNAF aerogels (400 W and 900 W) after smoothing of the measured spectra. The large scatter observed for the hybrid BNAF samples indicates ill-conditioned inversion, likely due to fluid–frame coupling and deviation from rigid-frame assumptions.

## **Table S1.** Reported sound absorption and sound transmission loss (STL) for representative aerogel-based acoustic materials.

| Material / system | Aerogel type | ρ (kg m⁻³) | t (mm) | Absorption (frequency range) | STL (frequency range) | Reference |
| --- | --- | --- | --- | --- | --- | --- |
| GO/PVA aerogel in Nomex honeycomb (GPA-1 series) | Graphene oxide / PVA aerogel | 2.1–7.4 | 25–37.5 | ȳα = 0.74–0.79 (400–2500 Hz) | ȳSTL up to 15.8 dB (400–2500 Hz) | Rapisarda et al., Sci. Rep. 11, 11656 (2021)^[2]^ |
| Modified GO/PVA aerogel (GOP series) | Graphene oxide / PVA aerogel | ≈ 6.5 | 25 | ȳα ≈ 0.72 (500–1500 Hz) | – | Rapisarda et al., ACS Appl. Mater. Interfaces (2022)^[3]^ |
| Polyimide aerogel (PIA-3%) | Polyimide aerogel | ≈ 33 | 20 | ȳα = 0.602 (250–6300 Hz) | ȳSTL = 11.94 dB (250–6300 Hz) | Wang et al., RSC Adv. 15, 23237 (2025)^[4]^ |
| Polyimide aerogel (PIA-6%) | Polyimide aerogel | ≈ 75 | 20 | ȳα = 0.416 (250–6300 Hz) | ȳSTL = 30.38 dB (250–6300 Hz) | Wang et al., RSC Adv. 15, 23237 (2025)^[4]^ |
| Polyurea aerogel | Polyurea aerogel | 250 | 5 | – | > 30 dB (1–4 kHz) | Malakooti et al., J. Non-Cryst. Solids 476, 36 (2017)^[5]^ |
| Silica aerogel / PET blanket | Silica aerogel composite blanket | 50–500 (typical) | 5–10 (typical) | Peak α often > 0.8 at mid–high frequencies (e.g. 1000–4000 Hz) | – | Talebi et al., Constr. Build. Mater. 220, 76 (2019)^[6]^ |

**SI References**

[1]	L. Jaouen, E. Gourdon, P. Glé, *The Journal of the Acoustical Society of America* **2020**, 148, 1998.

[2]	M. Rapisarda, G.-P. Malfense Fierro, M. Meo, *Scientific Reports* **2021**, 11, 10572.

[3]	M. Rapisarda, M. Meo, *ACS Applied Materials & Interfaces* **2022**, 14, 43646.

[4]	W. Wang, S. Wang, D. Li, J. Yang, H. Li, *RSC Advances* **2025**, 15, 42421.

[5]	S. Malakooti, H. G. Churu, A. Lee, T. Xu, H. Luo, N. Xiang, C. Sotiriou-Leventis, N. Leventis, H. Lu, *Journal of Non-Crystalline Solids* **2017**, 476, 36.

[6]	Z. Talebi, P. Soltani, N. Habibi, F. Latifi, *Construction and Building Materials* **2019**, 220, 76.
